# Molecular Docking and Virtual Screening of an Influenza Virus Inhibitor That Disrupts Protein–Protein Interactions

**DOI:** 10.3390/v13112229

**Published:** 2021-11-05

**Authors:** Yixin Ren, Sihui Long, Shuang Cao

**Affiliations:** Key Laboratory for Green Chemical Process of Ministry of Education, School of Chemical Engineering and Pharmacy, Wuhan Institute of Technology, Wuhan 430205, China; moondryad@outlook.com (Y.R.); sihuilong@wit.edu.cn (S.L.)

**Keywords:** RdRp, influenza virus, molecular dynamics, docking, virtual screening, protein–protein interactions

## Abstract

Influenza is an acute respiratory infection caused by the influenza virus, but few drugs are available for its treatment. Consequently, researchers have been engaged in efforts to discover new antiviral mechanisms that can lay the foundation for novel anti-influenza drugs. The viral RNA-dependent RNA polymerase (RdRp) is an enzyme that plays an indispensable role in the viral infection process, which is directly linked to the survival of the virus. Methods of inhibiting PB1–PB2 (basic polymerase 1–basic polymerase 2) interactions, which are a key part of RdRp enzyme activity, are integral in the design of novel antiviral drugs, a specific PB1–PB2 interactions inhibitor has not been reported. We have screened Enamine’s database and conducted a parallel screening of multiple docking schemes, followed by simulations of molecular dynamics to determine the structure of a stable ligand—PB1 complex. We also calculated the free energy of binding between the screened compounds and PB1 protein. Ultimately, we screened and identified a potential PB1–PB2 inhibitor using the ADMET prediction model.

## 1. Introduction

The influenza virus causes acute respiratory viral infections, which induce complications that often require hospitalization and may even lead to death. At the same time, the virus is easily transmissible from person to person and can infect individuals of all ages [1]. Consequently, seasonal influenza is deemed a serious public health concern.

Influenza viruses belong to the family of Orthomyxoviridae and are classified into four types: A, B, C, and D [2,3,4], and the first three types are able to infect humans. Influenza A virus (IAV) is prone to cause periodic pandemics because of the frequent mutation to escape the host immune system [5,6]. Influenza B virus (IBV) is relatively pathogenic to human beings, but it has not caused a global pandemic [2]. Influenza C virus (ICV) only causes mild infection, and affects public health slightly [7]. Influenza D virus (IDV) mainly infects pigs and cattle, but not human [8].

Current treatment options for tackling influenza viruses are limited, and drug resistance is a growing problem. The WHO recommends treatment with a neuraminidase inhibitor (oseltamivir) within 48 h of flu symptoms [9]. All circulating influenza viruses are resistant to adamantane drugs, such as amantadine and rimantadine [2,10,11]. According to the findings of the Chinese National Influenza Center’s resistance surveillance, all strains of influenza A/H1N1 and A/H3N2 viruses subtypes are resistant to amantadine analogues [10,11], and resistance to neuraminidase inhibitors has been reported in three cases, but the surveillance data revealed one case of resistance to neuraminidase inhibitors among influenza A/H1N1 viruses strains between 1 October 2019 and 13 September 2020 [12]. Therefore, the development of novel anti-influenza drugs is an urgent task.

Up to now, the antiviral drugs in the international market have been mainly divided into three categories: M2 ion channel blockers, neuraminidase inhibitors and RdRp drugs. Amantadine and Rimantadine are M2 ion channel blockers which block ion transport by binding to M2 ion channel proteins, thereby inhibiting viral replication and preventing the viral infection of new cells. Due to the S31N mutation [13,14], Amantadine cannot tightly fit with the ion channel, allowing the ions to pass through, and finally leading to drug resistance.

The neuraminidase inhibitors mainly include Zanamivir, Oseltamivir and Peramivir, which can mimic the natural substrate of NA, sialic acid, by blocking its active site and preventing it from catalyzing the hydrolysis of sialic acid, thus preventing the release of viral particles. Drug resistance is mainly due to the mutation of the binding site and the reduction of neuraminidase activity [15,16].

Recently developed anti-influenza drugs are mainly RdRp drugs that target the virus [17,18]. RdRp of influenza A virus consists of three different subunits: acidic polymerase, basic polymerase 1, and basic polymerase 2 (PA, BP1, and PB2), and antiviral effects can be achieved through the successful blocking of protein–protein interactions (PPIs) during protein assembly [19,20]. The currently marketed RdRp drugs include Favipiravir and Baloxavir. Favipiravir, as nucleoside analogues, is only used after the conventional antiviral drug treatment is ineffective, owing to its disadvantages of high toxicity, teratogenicity and abnormal behavior, etc. Baloxavir is an endonuclease inhibitor superior to neuraminidase inhibitors with a lower rate of adverse reactions. Reports indicate that the I38T/M/F mutation may lead to the development of resistance to Baloxavir. In recent years, small molecule inhibitors targeting PA–PB1 interactions have been extensively studied [21,22,23]. However, inhibitors targeting PB1–PB2 interactions have been rarely reported [18].

**Figure 1:**
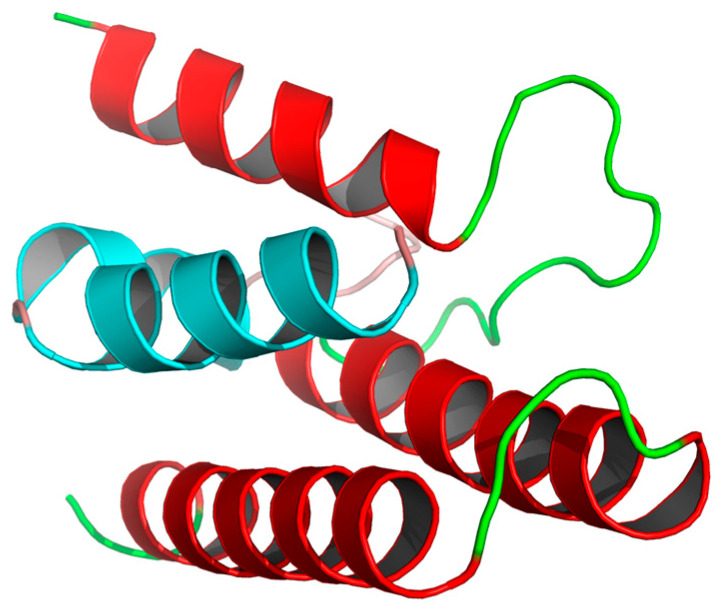
An overall ribbon diagram showing the structure of the complex, with helices from PB1 colored red, and helices from PB2 colored cyan. Coil regions are colored green.

In this study, we developed a novel PPI inhibitor via a computer-aided design based on PB1–PP2 interactions [24,25]. Figure 1 depicts the positions of interacting PB1 and PB2 (the red part is PB1, and the cyan part is PB2). The end portion of PB2 is wrapped in three α-helices at the C-terminal of PB1. An antiviral effect can be expected through the occupation of the PB1 pocket by small molecules that prevent the binding of PB1 and PB2.

To disrupt the interactions between PB1 and PB2, we examined key amino acids at their junction. For the PB1 fraction, Pooleal et al. characterized a polypeptide from the C-terminus of PB1, which disrupted the binding of PB1C to PB2N in experiments, thus inhibiting the activity of influenza RdRp and effectively inhibiting virus replication [26]. Monoclonal antibodies against the PB1 polypeptide of the influenza A virus can also interfere with viral mRNA synthesis in vitro. Moreover, the deletion and mutation of PB1 binding site residues can disrupt normal viral replication [27,28].

For the PB2 fraction, viral RNA polymerase activity was decreased through the deletion of 27 amino acids from the PB2N terminal [26,29]. These 27 amino acids, which are located at the PB2N terminal, can be applied as a chromogen reagent of viral transcription after binding to green fluorescent protein [30]. A monoclonal antibody developed against the influenza A virus PB2 peptide also interferes with PB1–PP2 binding, thereby inhibiting viral activity in vitro [31,32].

There is a high level of recognition specificity related to PB1 and PB2, and viral replication is inhibited by mutual loss, mutation, and substitution. Therefore, the disruption of the binding of these parts (PB1 731–757 and PB2 0–12) can effectively inhibit viral replication. We therefore designed small molecule inhibitors by replacing PB2.

In this study, we have designed a novel small molecule PPI inhibitor to occupy the PB1–PB2 interactions pocket with the aid of a computer design program. We subsequently conducted the virtual screening of Enamine PPI library containing 40,640 compounds, ultimately identified a molecule that could potentially serve as a lead compound.

## 2. Materials and Methods

The experimental procedure was performed with an Intel^®^ Xeon^®^ CPU E5-2650 0 @ 2.00 GHz (Intel, Santa Clara, CA, USA) processor, using a Windows 10 (Microsoft Corporation, Redmond, WA, USA) operating system and a 4 GB NVIDIA Quadro 2000 graphics card (Nvidia, Santa Clara, CA, USA). PyMOL (Schrödinger, Inc., New York, NY, USA) was used as a 3D visualization window [33].

### 2.1. Pretreatment of the PB1 and PB2 Proteins of the Influenza Virus

The X-ray crystallographic structure of RNA polymerase PB1–PB2 subunits [34] (PDB ID: 2ZTT, resolution: 2.10 Å, r-value free: 0.272) was downloaded from the Protein Data Bank [35]. We retained one of the PB1 chains for docking and replaced selenomethionine with methionine in the PDB file format. The further processing of proteins was conducted according to docking software requirements. Using the AutoDock Vina software (Scripps Research, San Diego, CA, USA) [36], we manually removed water molecules, ligands, and ions, hydrogenated and protonated each residue in the protein file using PyMOL [37], finally converted the PDB to a PDBPT file for subsequent docking using the Obabel program (University of Pittsburgh, Pittsburgh, USA) [38]. We applied the existing pre-processing function in the SYBYL software to remove water molecules, ligands, and ions from the protein file and to hydrogenate each residue. We also used this to repair end residues, reinsert position files, repair residues, and minimize energy and possible inter-atomic collisions. We used the protein preparation tool in the Discovery Studio software to complete the process of removing water molecules, ligands, and ions, hydrogenated and protonated each residue in the protein file.

### 2.2. Ligand Preparation

We selected Enamine’s database, which provides a specific molecular library for PPI screening [39]. This database was selected because the molecules within this collection have an α-helix-like structure, with the ability to mimic protein motifs. The compounds in the database can be purchased directly for testing after completion of the screening process. The molecular library was split using the OpenBabel program and was subsequently hydrogenated, and energy minimized using a high-precision MMFF94 force field [38]. The 3D structures ultimately generated were used in the molecular docking studies.

### 2.3. Molecular Docking

**Figure 2:**
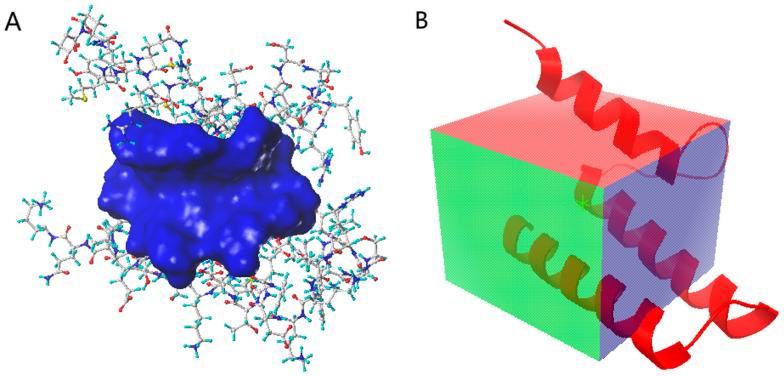
(**A**) The docking pocket for SYBYL and Discovery Studio programs, blue: pocket; stick: protein; (**B**) the docking pocket for AutoDock Vina software, box: pocket; red: protein.

To select the docking pocket (Figure 2), part of the PB2 short peptide (0–12), which plays a major role in PPI, was selected as the simulated binding site using the SYBYL and Discovery Studio programs, and the amino acid space within 5 Å of the short peptide was selected as the docking pocket. A cube centered at x = 4.639, y = 7.639, and z = 5.861 with the side lengths of x, y, and z set respectively at 18.0, 22.0, and 22.0 was depicted with the AutoDock Vina software as a docking pocket.

The database molecules were docked into the docking pockets of PB1 protein using the Surflex-Dock Screen provided in the Sybyl 2.0 software. The docking results were scored and ranked, and 2000 compound conformations with high scores were selected for further docking. These molecular conformations were docked again to the active site by applying three modes of docking: the Surflex-Dock GeomX mode in the SYBYL 2.0 software, the LibDock mode in the Discovery Studio software (3dexperiencem, Aachen, Germany), and the AutoDock Vina software. Three molecules were selected for further study according to their docking positions relating to the receptor, considered in combination with their scores.

### 2.4. Simulation of Molecular Dynamics

Molecular dynamics (MD) simulations were performed using the docking results to enable the further analysis of the binding of protein–ligand complexes. All MD simulations were performed using the GROMACS package (University of Groningen, Groningen, Netherlands), version 2020.3 [40,41,42]. The AMBER99SB-ILDN force field was used to optimize proteins [43], and water molecules were modeled with TIP3P [44,45]. We used ACPYPE to calculate ligand charges and generate topology files for GAFF force [46]. The MD box was set up as a cube with the distance between each atom and the box exceeding 0.8 nm. SOL water was added to the box at a density of 1000 g/L. Chloride ions were used to replace water molecules in the system to achieve an electrically neutral simulation system. The relaxation system was then simulated using the Verlet algorithm for energy minimization to eliminate the overlap between atoms and avoid problems such as the overly close proximity of homogeneous charges [47]. The system was subjected to a 100 ps restricted kinetic simulation at 298.15 K. Lastly, the regular dynamics of the system were simulated using the Verlet algorithm [47]. The integration step was set at 0.002 ps, and simulations were conducted for 10 ns in total. The simulations were performed in an isothermal isobaric regime at 298.15 K and under 1 bar pressure, with temperature and pressure respectively controlled with the V-rescale and Parrinello–Rahman methods [48], and PBC (periodic boundary condition) was enabled. The RMSD (root mean squared deviation) was calculated for protein–protein and protein–small molecules interactions. MD trajectories were viewed using VMD software (University of Illinois at Urbana–Champaign, Urbana, IL, USA) [49].

### 2.5. Calculation of the Binding Free Energy

The binding free energy between the protein–ligand complexes was estimated using the MM/PBSA equation [50]. The APBS lattice parameters are output according to the MD results, and the APBS software (Pacific Northwest National Laboratory, Richland, DC, USA) [51] was applied to calculate polar solvation energy (PB) and polar solvation energy (SA). Subsequently, the MM contribution was calculated, and the energy decomposition of the residues was performed.

The MM energy was calculated on the basis of directly accumulated potential, taking into account the dielectric constant of the solute (protein). PB energy was calculated by subtracting the energy of the aqueous phase from the energy in the vacuum. SA energy was calculated by multiplying the solvent-accessible surface area of each atom by the surface tension.

### 2.6. Binding Force Analysis

We used the Discovery Studio software to analyze the final results and to examine the non-bonding interactions and hydrogen bonding between the ligand and the receptor.

The non-bonding interactions are the main outlets to examine the hydrophobic interactions between π ring and alkyl. With respect to the geometry of π–π stacked interactions, the distance between the centroid of each π ring pair is less than 6 Å. At least one pair of atoms on both rings is less than 4.5 Å. The angle between the normal of one or both rings and the centroid-centroid vector must be between 0° and ±50°, and the angle between the normal to each ring must be between 0° and ±35°.

With respect to the geometry of alkyl interactions, the ligand hydrophobic groups are not attached to charged atoms and have a surface area greater than 0.65 times the surface area of the methyl group. Moreover, the distance between the centroid of different hydrophobic groups on the receptor and ligand is less than 4.5 Å.

With respect to the geometry of π-alkyl interactions, the hydrogens acting as the donor must be connected to a nonaromatic carbon atom. The distances between the hydrogen and the center of the π ring must be less than 4.1 Å. The C-H-centroid angle cannot deviate from linearity by more than 20° and the angle between the C-centroid vector and the normal to the ring plane is not more than 45°.

With respect to the geometry of hydrogen bond definition, the distance between the hydrogen atom and the hydrogen bond receptor was less than 2.7 or the distance between that hydrogen bond donor and the hydrogen bond receptor is less than 3.4. At the same time, the angle formed between the hydrogen atom–hydrogen bond donor–hydrogen bond receptor should be less than 35°.

### 2.7. ADMET Prediction

We used the ADMET Descriptors module and the TOPKAT module in Discovery Studio software to predict the ADMET and toxicological properties of the compounds.

The ADMET Descriptors module is developed based on descriptors computed by linear formulas. The water-soluble model was developed using a training set of 775 compounds and a test set of 34 compounds using descriptors of solubility and solubility levels (R^2^ = 0.88, SD = 0.79). Blood–brain permeability was developed using a training set of 102 compounds and a test set of 86 compounds. Descriptors used were 2D polar surface area, halogen content, and LogP (R^2^ = 0.88, SD = 0.31). A toxicity model was generated by modified Bayesian learning. A leave-one-out cross-validation was conducted. Model sensitivity is 0.816, specificity is 0.854, and concordance rate is 0.834.

Based on the 2D molecular structure, TOPKAT module resolved the predicted compound into functional groups and substructures, and converted them into descriptors, such as two-dimensional molecular, electronic and spatial descriptors, by similarity scoring function. Finally, the descriptors were brought into the different structural toxicity relationship equations of Quan ta va in TOPKAT module to calculate the possible toxicity values of the corresponding compounds.

## 3. Results

### 3.1. Protein Preparation

Only 2ZTT and 3A1G have been reported for the crystal structure of RNA polymerase PB1–PB2 subunit [34]. The two protein structures have resolutions between 1.5 Å and 2.5 Å. A Ramachandran plot prediction of the two proteins was generated (Table 1). Most of the 2ZTT residues were favorably located (96%); a few residues were located in allowed areas (4%), and no residues were outliers. In the case of 3AIG, most of the residues were favorably located (98%), a few residues were in allowed locations (2%), and one residue was an outlier. For this screening, 2ZTT was selected as the docked protein structure.

**Figure 3:**
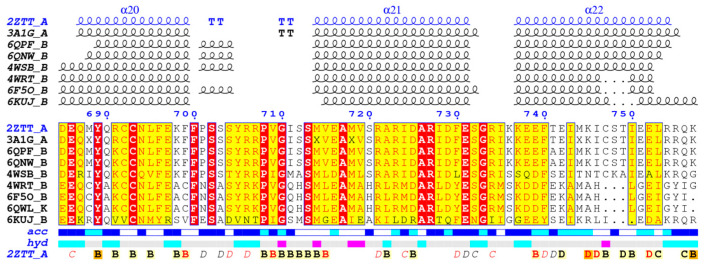
Comparison of the results of A-chain sequences of 2ZTT.

To ensure the generalizability of the screening results, we used the ENDscript/ESPript software to calculate the conservativeness of docked proteins among homologous proteins [52]. As shown in Figure 3, among the homologous proteins, the PB1C-terminal protein of the three α-helices was well conserved sequence comparison has been completed by Clustal Omega web server [53]. The inhibitor designed based on the protein binding site can effectively inhibit different subtypes of influenza A virus and influenza B virus (4WRT, 6F5O and 6QWL). There are some differences (6KUJ) in the corresponding residues of influenza D virus, which may lead to the decrease of activity. The binding site of influenza C virus is too different, so there is no universality.

Protein pretreatment: given the lack of non-standard amino acid force fields in the docking software, selenomethionine was manually replaced with methionine, and irrelevant atoms were removed during MD simulations and molecular docking. Terminal residues in the protein structure were repaired, hydrogens were added, force field files were reincorporated, and energy minimization of the protein was conducted.

### 3.2. Molecular Docking

Initially, we selected the top 2000 compounds for fine docking, on the basis of the Sybyl docking results. These molecules were docked to the active site using three modes of docking to enable a precise evaluation of the binding. The docking results for the top 200 compounds are shown in Table 2.

**Figure 4:**
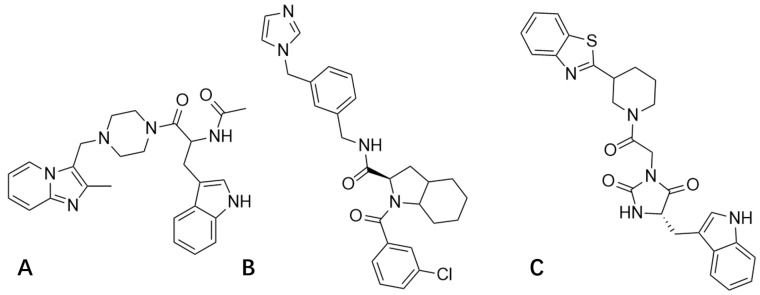
Compound 3206 (**A**); Compound 4808 (**B**); and Compound 15144 (**C**).

Statistical advantages in docking scores were identified for the following compounds: 3206 (Sybyl: 7.3773; Vina: −7.8; Discovery Studio: 109.4), 4808 (Sybyl: 7.5041; Vina: −7.7; Discovery Studio: 110.254), and 15144 (Sybyl: 7.8586; Vina: −7.7; Discovery Studio: 109.751) (Figure 4).

### 3.3. Molecular Dynamics

**Figure 5:**
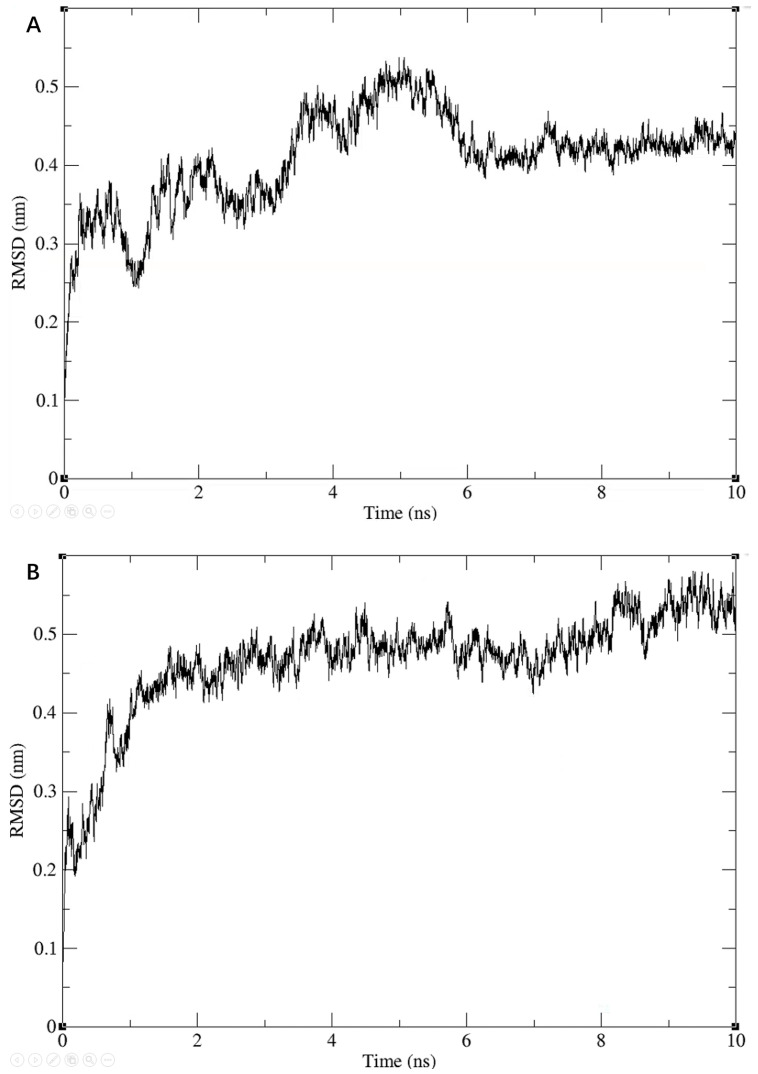

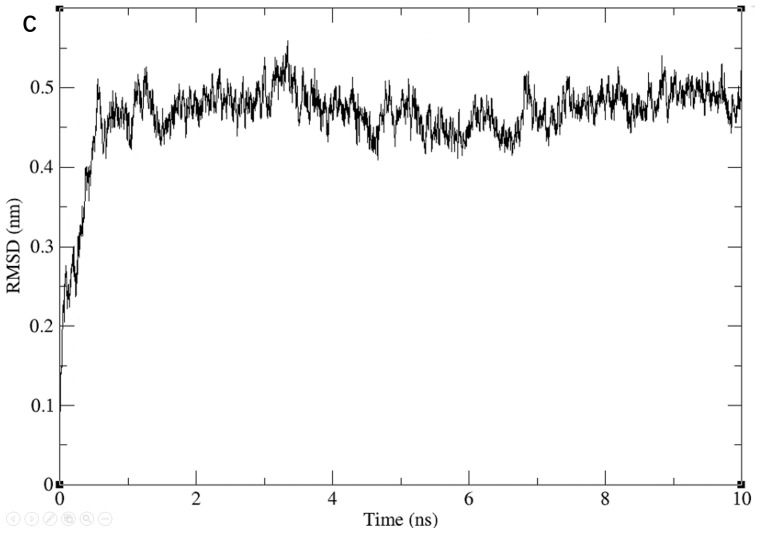
Results of the 10 ns molecular dynamic simulations for three ligand-2ZTT complexes. RMSD of Compound 3206 with protein (**A**); RMSD of Compound 4808 with protein (**B**); RMSD of Compound 15144 with protein (**C**).

We further checked the conformational plausibility of the screened compounds by performing MD simulations. Specifically, we performed MD simulations for three molecules for 10 ns each. During the simulations, the RMSD values of the entire system showed a gradual convergence, and eventually became stable, as shown in Figure 5.

### 3.4. Calculation of the Binding Free Energy

After we obtained stable protein–ligand complexes through MD simulations, we calculated the binding energy between the three compounds and the protein using the MM/PBSA equation (Table 3).

As shown in Table 3, the values of the binding free energies of all three systems were below zero, indicating that the binding of protein–ligand complexes was stable. The binding free energies of Compounds 3206 and 4808 were, respectively, −128.049 kJ/mol and −122.761 kJ/mol, which exceeded that of Compound 15144 (−106.93 kJ/mol). Van der Waals interaction accounted for the binding of Compounds 3206 and 4808 with protein, and the electrostatic interaction of these two compounds, had particularly different of −39.663 kJ/mol and 1.721 kJ/mol. These results suggest that compound 4808 is more suitable for use as a lead inhibitor by changing the molecular polarity or local polarity to facilitate increased compound–protein interactions.

The molecular dynamics simulation of the polypeptide–protein complex was performed for 10 ns with the polypeptide from the N-terminus of PB2 as ligands. Considering the macromolecules as the comparison object, the Gibbs free energy was used as the comparison standard. The Gibbs free energy of polypeptide and PB1 was −34.792 kJ/mol comparing with −45.007 kJ/mol of compound 4808 and PB1, which indicated that the screened molecules could inhibit PB1–PB2 interactions effectively and competitively.

**Figure 6:**
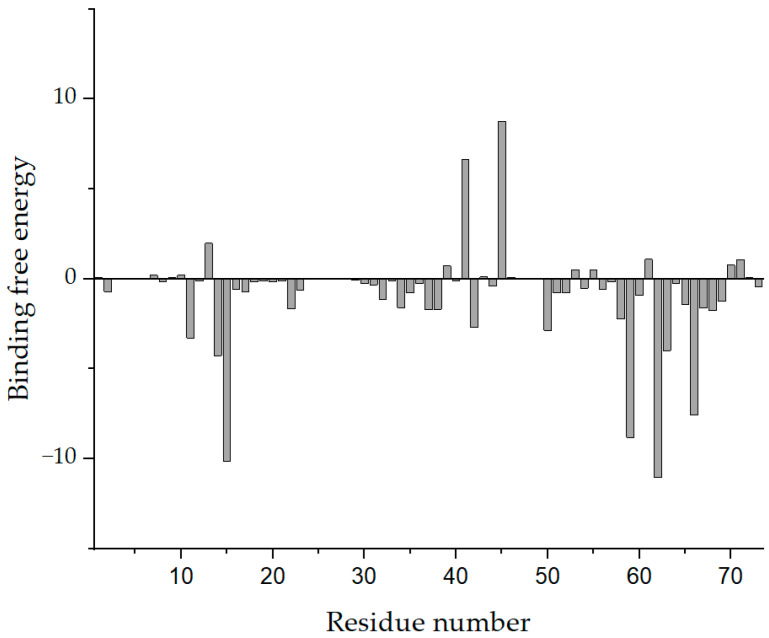
Analysis of the binding free energy decomposition of Compound 4808.

Subsequently, we performed a binding free energy decomposition analysis of Compound 4808; the results are depicted in Figure 6.

The results of the analysis of residue decomposition indicated that protein residues 39, 41, and 45 (residue contributions of 0.425, 6.956, and 8.779 kJ/mol, respectively) had an unfavorable impact on the binding. Residues 15, 59, 62, and 66 (residue contributions of −10.113, −8.797, −11.035, and −7.599 kJ/mol, respectively) were the main contributors to the binding of the protein to Compound 4808.

### 3.5. Binding Force Analysis

**Figure 7:**
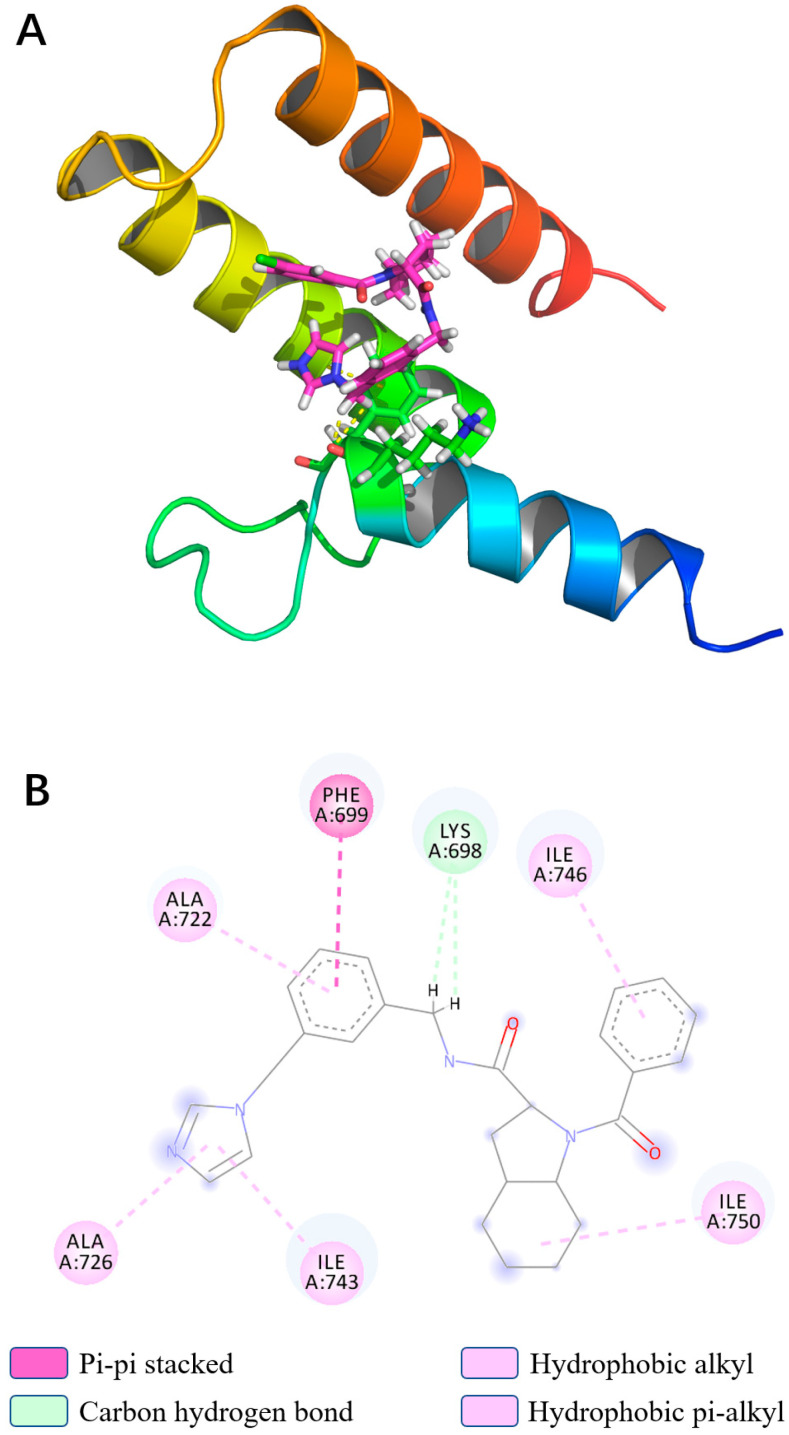
(**A**) Interaction ribbon diagram, magenta: compound 4808; ribbon: PB1 protein; yellow: hydrogen bond; (**B**) two-dimensional protein–ligand contacts of compound 4808 predicted through molecular dynamics.

In light of the results of the MD simulations, we performed a binding force analysis using Discovery Studio’s receptor–ligand interaction calculation tool (Figure 7). The interactions between protein and Compound 4808 mainly comprised hydrogen bonding and hydrophobic interactions. For the hydrogen bond interactions, residue 14 formed two carbon–hydrogen bonds with bond lengths of 2.509 Å and 2.534 Å. The following hydrophobic interactions occurred: a π–π stacking interaction between residue 15 and the compound; an alkyl interaction between residue 66 and the compound; and π–alkyl interactions between residues 38, 42, 59, and 62 and the compound.

### 3.6. ADMET Predictions

ADMET predictions indicated that Compound 4808 is slightly soluble in water at 25 °C (log (SW) = −5.137). A brain–blood ratio between 1:1 and 1:5 does not inhibit cytochrome P450 2D6. The Bayesian score for pigment toxicity was −2.63393, which is associated with some toxicity to the liver. The Bayesian score for hepatotoxicity was −1.85674, which is associated with adequate intestinal absorption (ADMET_Absorption_T2_2D < 6.1261).

Toxicity predictions were calculated using the Ames heteroaromatic model. According to the results of the prediction study conducted on PB1–PB2 protein binding, the computed probability of mutagenicity for Compound 4808 was 0.000 (discriminant score = −29.801). The computed rat oral lethal dose 50 (LD_50_) value for Compound 4808 obtained with the Rat Oral LD_50_ heteroaromatic model was 111.8 mg/kg. The lower and upper 95% confidence limits were 13.5 mg/kg and 925.8 mg/kg, respectively, and the computed rat oral LD_50_ log value (1/Moles) was 3.630. These parameters were checked to be within their standard ranges and compound 4804 is suitable for further development as a lead compound.

## 4. Conclusions

Seasonal influenza is considered to be a serious public health problem. Drug resistance to existing influenza drugs requires researchers to develop new anti-influenza mechanisms. PB1–PB2 interactions are very important for the formation of RdRp, which is a key structural component of virus replication, so it is an attractive target for drug development. To date, no specific inhibitor of PB1–PB2 interactions has been reported. In this study, our main goal was to obtain a lead compound with drug-forming potential through a virtual screen. Firstly, according to the analysis of the Ramachandran diagram, protein 2ZTT is chosen as the docked protein structure. A pocket of PB1 protein, which currently has no drug development in this pocket, was selected as a target for drug design. In order to ensure a low false positive of the screened drug, we chose a compound library targeting PPI inhibitors and a cross-validation scheme using three software tools for docking. Finally, three compounds were screened out through molecular docking. In order to further ensure the effectiveness of screening, molecular dynamics simulation was performed for 10 ns. The simulations results show that these compounds could be stably bound in PB1 pocket, and the binding effect is more than 100 kJ/mol. We chose compound 4808 as the screening result, and analyzed the free energy decomposition of compound 4808. Based on the decomposition results, it is possible to improve drug activity by reducing the bad contact with protein residues 30, 41 and 45, and further enhancing the binding effect of residues 15, 59, 62 and 66. However, before entering clinical practice, further work still needs to be done on PB1–PB2 inhibitors. For example, there are few co-crystallization studies on PB1–PB2 inhibitors. The activity of them only reaches the micromolar level, and further structural optimization is required. This study provides a lead compound for developing new targets of antiviral drugs, so as to solve the drug resistance problem of current anti-influenza drugs, and puts forward suggestions for improving the compound. However, the compound was only computationally compared with existing active compounds, and further studies are needed to obtain in vivo activity test data.

## Figures and Tables

**Table 1:** Results of the Ramachandran plot analysis of 2ZTT and 3A1G.

PDB	Favored	Allowed	Outliers
2ZTT	201 (96%)	9 (4%)	0 (0%)
3A1G	209 (98%)	4 (2%)	1 (0%)

**Table 2:** Scores obtained for the three docking schemes.

Compound	Score (Sybyl)	Binding Energies (Vina)	Score (Discovery Studio)
Compound 6320	5.4247	−8.5	100.215
Compound 13815	6.4074	−8.1	100.972
Compound 8770	6.6121	−8.1	91.1954
Compound 979	6.2028	−7.9	103.837
Compound 15672	6.2651	−7.9	101.386
Compound 11902	7.0193	−7.8	117.427
Compound 7679	6.3579	−7.8	111.038
Compound 4804	6.4706	−7.8	110.254
Compound 3206	7.3773	−7.8	109.4
Compound 637	6.0599	−7.8	104.964
Compound 5068	6.1561	−7.8	104.127
Compound 17948	6.3089	−7.8	104.063
Compound 13816	5.2263	−7.8	100.972
Compound 3165	7.8442	−7.8	100.56
Compound 6322	5.6455	−7.8	99.0563
Compound 12538	6.5092	−7.7	120.272
Compound 4808	7.5041	−7.7	110.254
Compound 15144	7.8586	−7.7	109.751
Compound 8836	6.4969	−7.7	105.816
Compound 15026	6.6115	−7.7	105.675
Compound 11365	7.7766	−7.7	105.223
Compound 4560	6.5334	−7.7	103.375
Compound 11915	6.8264	−7.7	103.172
Compound 8721	7.5264	−7.7	101.89
Compound 6158	6.5414	−7.7	100.276
Compound 13727	6.8292	−7.7	97.5998
Compound 5201	6.4911	−7.7	97.0757
Compound 12269	6.0082	−7.7	96.9189
Compound 1451	5.4295	−7.7	96.7586
Compound 118	5.4985	−7.7	92.4782
Compound 11904	7.6596	−7.6	117.427
Compound 8631	5.9089	−7.6	111.328
Compound 943	6.0759	−7.6	110.615
Compound 7267	6.469	−7.6	108.918
Compound 13884	6.6642	−7.6	108.747
Compound 9555	7.4359	−7.6	106.817
Compound 9561	7.1219	−7.6	106.817
Compound 19179	5.9982	−7.6	105.181
Compound 13047	6.6693	−7.6	104.76
Compound 9599	6.2425	−7.6	103.66
Compound 4108	5.6692	−7.6	101.636
Compound 6159	6.5045	−7.6	100.276
Compound 10747	6.4367	−7.6	99.8972
Compound 10746	6.3898	−7.6	99.8972
Compound 1481	7.3196	−7.6	99.869
Compound 13726	5.6056	−7.6	99.346
Compound 9150	5.5108	−7.6	98.0525
Compound 5609	6.8803	−7.6	97.5804
Compound 12672	6.9693	−7.6	97.5469
Compound 8636	7.6168	−7.6	97.3231
Compound 8632	6.4931	−7.5	111.328
Compound 10818	6.6349	−7.5	110.749
Compound 2100	7.1846	−7.5	110.593
Compound 5242	6.8628	−7.5	110.566
Compound 6845	5.7634	−7.5	109.107
Compound 7266	5.8177	−7.5	108.918
Compound 13888	6.9957	−7.5	108.747
Compound 9012	7.4817	−7.5	108.074
Compound 1095	6.5534	−7.5	105.985
Compound 1832	7.5959	−7.5	103.958
Compound 2510	8.1886	−7.5	103.477
Compound 1947	6.3243	−7.5	101.659
Compound 8626	5.7178	−7.5	100.117
Compound 5611	6.3159	−7.5	98.6665
Compound 104	6.3199	−7.5	98.2362
Compound 105	6.2256	−7.5	98.2362
Compound 9149	5.8438	−7.5	98.0525
Compound 10315	6.0586	−7.5	97.5558
Compound 10316	5.8198	−7.5	97.5558
Compound 9392	5.9734	−7.5	97.3548
Compound 7142	5.2493	−7.5	97.0345
Compound 15055	7.1738	−7.5	95.3654
Compound 6657	5.2667	−7.5	93.2045
Compound 8194	5.5902	−7.5	91.8273
Compound 15053	6.5572	−7.5	90.1132
Compound 2289	6.4862	−7.4	113.683
Compound 7678	5.6392	−7.4	111.038
Compound 2170	8.3252	−7.40	110.926
Compound 9481	7.9091	−7.4	108.32
Compound 15029	6.2396	−7.4	105.675
Compound 11630	5.4344	−7.4	104.942
Compound 13048	7.7122	−7.4	104.76
Compound 13049	7.67	−7.4	104.76
Compound 8224	5.02	−7.4	104.605
Compound 1831	7.7449	−7.4	103.958
Compound 1058	6.2938	−7.4	103.642
Compound 2638	6.1372	−7.4	102.785
Compound 3836	5.926	−7.4	102.65
Compound 2950	5.833	−7.4	99.3493
Compound 7656	6.9155	−7.4	98.4305
Compound 120	5.7108	−7.4	97.1121
Compound 10134	6.1476	−7.4	96.6006
Compound 1242	5.4844	−7.3	109.72
Compound 3813	7.5651	−7.3	107.587
Compound 12305	5.3583	−7.3	98.7621
Compound 2046	6.6877	−7.3	98.6504
Compound 5098	7.0632	−7.3	98.1682
Compound 11824	5.3915	−7.3	98.022
Compound 11612	7.2281	−7.3	94.3189
Compound 2158	7.3805	−7.3	91.2644

**Table 3:** Results of the MM/PBSA calculation.

Compound No.	Binding Free Energy	PB Energy	SA Energy	Electrostatic Attraction	Van der Waals Force
3206	−128.049	89.613	−21.492	−39.663	−156.507
4808	−122.761	62.573	−20.492	1.721	−166.564
15144	−106.93	30.743	−16.627	−9.602	−111.444

## Data Availability

The data presented in this study are available on request from the corresponding author.
